# In the Eye of the Covid-19 Storm: A Web-Based Survey of Psychological Distress Among People Living in Lombardy

**DOI:** 10.3389/fpsyg.2021.566753

**Published:** 2021-02-24

**Authors:** Emanuela Saita, Federica Facchin, Francesco Pagnini, Sara Molgora

**Affiliations:** Department of Psychology, Catholic University of the Sacred Heart, Milan, Italy

**Keywords:** COVID-19, anxiety, depression, perceived stress, online survey, Lombardy (Italy)

## Abstract

In March 2020, the World Health Organization announced the Covid-19 outbreak a pandemic and restrictive measures were enacted by the Governments to fight the spread of the virus. In Italy, these measures included a nationwide lockdown, with limited exceptions including grocery shopping, certain work activities, and healthcare. Consistently with findings from previous studies investigating the psychological impact of similar pandemics [e.g., Severe Acute Respiratory Syndrome (SARS)], there is evidence that Covid-19 is associated with negative mental health outcomes. Given this background, we conducted a cross-sectional study aimed at investigating the impact of the Covid-19 pandemic and the subsequent restrictive measures imposed by the Government on the psychological health of Italian men and women aged = 18 years and living in Lombardy, one of the worst-hit regions. The study also aimed at identifying what factors are associated with specific psychological outcomes. Thus, we developed an online survey that included a researcher-made questionnaire to collect sociodemographic, household, general health, and pandemic-related information. The Generalized Anxiety Disorder-7, the Patient Health Questionnaire-9 and the Perceived Stress Scale were used to assess anxiety, depression, and perceived stress, respectively. We found that younger age, greater concerns about the pandemic, female gender, being unmarried, not having children, and being a student were associated with worse psychological health. These findings may provide further insight into the risk factors associated with negative psychological outcomes during the current pandemic, with identification of vulnerable groups. This body of evidence may help professionals implement targeted psychosocial treatment and prevention programs.

## Introduction

Since December 2019, when the first cases were reported in the city of Wuhan, China, the Covid-19 outbreak has spread worldwide, facilitated by the contagiousness of asymptomatic individuals, as well as by international travels (Matias et al., 2020; Vigo et al., 2020). In the morning of 12 March 2020, with more than 20,000 confirmed cases and almost 1,000 deaths in Europe, the World Health Organization (WHO) announced the Covid-19 outbreak a pandemic^1^. Different types of restrictive measures were enacted by the Governments to contain the spread of the disease. In Italy, these measures involved a nationwide lockdown from 9 March to 4 May 2020, with exceptions allowed only for medical reasons and for necessities like grocery shopping and work.

The negative psychological impact of similar pandemics [such as Severe Acute Respiratory Syndrome (SARS), Ebola, the 2009–2010 H1N1 influenza, Middle East Respiratory Syndrome (MERS), and equine influenza] has been highlighted in previous studies, whose findings were recently summarized by Brooks et al. (2020). In their rapid literature review, the authors included 24 studies reporting evidence on the psychological consequences of quarantine, which entails an overall high prevalence of psychological distress, sense of isolation, anxiety, mood disorders, insomnia, anger and frustration, and even post-traumatic stress disorder (Brooks et al., 2020). Besides the fact that viral outbreaks represent a severe threat to people’s lives, the adverse psychological effects of pandemics such as Covid-19 also derive from the consequent economic crisis, with millions of people left out of work or at risk of losing their job (Vigo et al., 2020). For all these reasons, the psychological burden of pandemics has been referred to as a “parallel epidemic” (Yao et al., 2020).

In a study focused on the immediate psychological reactions displayed by the Chinese population during the initial stage of the Covid-19 outbreak (Wang C. et al., 2020), 54% of 1,210 respondents rated the psychological impact of the situation as moderate or severe, with depressive and anxiety symptoms reported by 16 and 29% of participants, respectively. Moreover, 75% of the participants were worried about their family members contracting the disease and were satisfied with the available health information. Risk factors associated with worse psychological conditions were female gender, student status, presence of physical symptoms such as myalgia, dizziness, coryza, and overall poor self-related health status, while appropriate preventive measures (such as hand washing and wearing a mask) and detailed health information were associated with better psychological outcomes.

In another study (Wang H. et al., 2020), younger, unmarried individuals, with poor social support, reported higher psychological distress than the rest of the sample. People with pre-existing physical and mental disorders (including substance abuse) represent a particularly vulnerable group due to the psychological burden of the pandemic, as well as to disruptions in their care (Vigo et al., 2020). Exposure to Covid-19 news can also influence the psychological impact of the disease by increasing stress and depressive symptoms, especially in individuals who report a high perceived vulnerability to the virus (Olagoke et al., 2020; Yao et al., 2020).

Given this background, more research is needed to further clarify the psychological impact of Covid-19, including risk and protective factors. In this regard, this study aims to examine the psychological consequences of the pandemic in Lombardy, the worst-hit Italian region (Odone et al., 2020). Specifically, our goal was to investigate the association between sociodemographic, household, general health, beliefs and concerns about the pandemic, and the psychological health of the community, with a specific focus on anxiety and depressive symptoms, and perceived stress.

## Materials and Methods

The study used a cross-sectional design, with data collected from 13 April to 10 May 2020 using an online survey that was delivered through the Qualtrics suite (Qualtrics, Provo, UT). Participants were recruited using a snowball sampling procedure, which also involved posting the invitation to participate in the research on social media. We included only participants aged ≥18 years, resident in Lombardy, and fluent in Italian. The study was approved by the Ethics Commission of the Department of Psychology of Università Cattolica del Sacro Cuore. Before completing the questionnaires, all participants provided electronic informed consent.

### Measures

A researcher-made questionnaire was developed to collect sociodemographic data (age, level of education, employment and marital status, and presence of children), household information (number of people in the same house, presence of pets, size of the house, and presence of garden or balcony), general health status (diagnosed physical or psychological conditions), and Covid-19 related information [Covid-19 diagnosis, concerns about the pandemic (e.g., “To what extent are you concerned about this pandemic?”; 1 = not at all, 4 = extremely), perceived risk for themselves and their significant others (e.g., “To what extent do you perceive yourself at risk due to the pandemic?”; 1 = low risk, 3 = high risk), fear of being infected or infecting others (e.g., “To what extent are you concerned about being infected by others?”; 1 = not at all, 5 = very much), and satisfaction with the information provided by public authorities (i.e., “To what extent are you satisfied with the quality of the information provided by public authorities?”; 1 = not at all, 5 = very much)]. The Italian version of three standardized self-report questionnaires was then used to assess participants’ psychological health: (1) the *Generalized Anxiety Disorder-7* (GAD-7; Spitzer et al., 2006; Bruno et al., 2020); (2) the *Patient Health Questionnaire-9* (PHQ-9; Kroenke et al., 2001; Mazzotti et al., 2003); (3) the *Perceived Stress Scale* (PSS; Cohen, 1994; Mondo et al., 2019).

The GAD-7 is a 7-item measure that allows the rapid detection of generalized anxiety disorder (GAD). Participants are asked to rate on a 4-point Likert scale (0 = not at all; 3 = nearly every day) how often they have been bothered by anxiety symptoms in the past 2 weeks. The global score ranges from 0 to 21, with higher scores indicating greater GAD. Scores of 10 or higher indicate possible clinically significant conditions (Spitzer et al., 2006).

The PHQ-9 is a widely used 9-item questionnaire for the screening of depression in non-psychiatric settings. The PHQ-9 detects the presence of a wide range of depressive symptoms (such as anhedonia, depressed mood, trouble sleeping, tiredness, and even suicidal thoughts) based on their frequency in the last 2 weeks, which is rated on a 0–3 Likert scale (0 = not at all; 3 = nearly every day). The PHQ-9 total score ranges from 0 to 27, with greater scores indicating worse psychological conditions. Similarly to the GAD-7, scores ≥10 indicate clinical cases (Gilbody et al., 2007).

The PSS is a 10-item questionnaire for assessing the perception of stress. Specifically, participants are asked to rate on a 0–4 Likert scale (0 = never; 4 = very often) how often they felt upset, nervous, unable to control and to cope with things in their life, angered, and overwhelmed, focusing on the last month. After reversing four positively stated items, all items are summed to obtain a total score that ranges between 0 and 40, with higher scores indicating greater perceived stress. Scores ranging from 0 to 13, 14 to 26, and 27 to 40 indicate low, moderate, and high stress, respectively (Cohen, 1994).

In this study, all these scales showed good internal consistency (Cronbach’s α was 0.894 for the GAD-7, 0.830 for the PHQ-9, and 0.925 for the PSS).

### Statistical Analyses

Once obtained the descriptive statistics, we examined whether the scores of the GAD-7, the PHQ-9, and the PSS-10 were normally distributed, considering skewness and kurtosis (–1/ + 1 was established as the acceptable range for normality). Given that these variables were not normally distributed, and considering that our analytic strategy involved comparisons between unbalanced groups, statistical analyses were performed using a non-parametric approach. Specifically, relations between continuous variables (such as for instance age and psychological symptoms) were explored using Spearman’s correlation. Group comparisons were conducted using the Mann-Whitney *U*-test or the Kruskal-Wallis *H*-test, as appropriate. Because our analytic strategy involved multiple comparisons, *Ps* < 0.01 were considered statistically significant. All statistical analyses were performed using the software SPSS, version 25.

Statistical power was computed based on previous data collected by our Department (Pagnini et al., 2020) using a conservative approach, suggesting a correlation between worries and well-being around 0.182. Under these circumstances, a sample of 313 participants would allow a power of 0.90. The power analysis was conducted using the software G^∗^Power 3.1 (Faul et al., 2009).

## Results

Our participants were 319 residents of Lombardy aged between 18 and 78 years old (*M* = 42.95; *SD* = 16.85). Most participants were women (81% vs. 19% of men). The characteristics of the sample based on the information collected using the researcher-made questionnaire (i.e., socio-demographic, household, health-related, and pandemic-related information) are reported in Table 1, while median, means and standard deviations for psychological health assessed using the GAD-7, the PHQ-9, and the PSS-10 are presented in Table 2. Considering the cut-offs of these three questionnaires, we found that 18.5% of the participants reported clinically significant anxiety, 17.6% had clinical depression (with 12.2% of participants having clinically significant symptoms of both anxiety and depression), while moderate levels of perceived stress were reported by most participants (96%).

**TABLE 1:** Socio-demographic, household, and pandemic-related information, and general health status.

Type of information		
**Socio-demographic information**		**%**
Level of education	Primary/elementary school	0.6
	Middle school	5.6
	High school	50.8
	University (bachelor’s degree)	14.7
	University (master’s degree)	18.8
	Doctoral degree	9.5
Occupational status	Full-time worker	35.4
	Part-time worker	8.5
	Self-employed	15.0
	Student with part-time job	6.0
	Student	13.8
	Retired	10.0
	Home-maker/housewife	6.6
	Unemployed	4.7
Marital status	Unmarried	40.9
	Married/cohabitating	51.3
	Separated/divorced	6.9
	Widowed	0.9
Presence of children	No	49.5
	Yes	50.5
**Household information**		**%**
House size	≤50 m^2^	2.3
	51–100 m^2^	50.5
	101–120 m^2^	14.6
	121–150 m^2^	12.7
	≥150m^2^	19.9
Presence of a balcony	No	11.7
	Yes	88.3
Presence of a garden	No	51.7
	Yes	48.3
Presence of pets	No	54.9
	Yes	45.1
**Pandemic-related information**		
Worries about the pandemic	(Four-point Likert scale)	M (*SD*) 3.18 (0.59)%
	Not worried at all	1.3
	Slightly worried	6.3
	Moderately worried	66.1
	Extremely worried	26.3
Risk perception	(Three-point Likert scale)	M (*SD*) 1.92 (0.66)%
	Low risk	26.3
	Moderate risk	55.5
	High risk	18.2
Concern about being infected by others	(Five-point Likert scale)	M (*SD*) 3.14 (0.82)
Concern about infecting others	(Five-point Likert scale)	M (*SD*) 3.26 (1.09)
Diagnosed with Covid-19	No	%57.4
	Yes	2.8
	I don’t know	39.5
	I prefer not to answer	0.3
Family members diagnosed with Covid-19	No	61.5
	Yes	7.0
	I don’t know	31.5
	I prefer not to answer	–
Friends or coworkers diagnosed with Covid-19	No	32.9
	Yes	40.4
**Socio-demographic information**		**%**
	I don’t know	26.4
	I prefer not to answer	0.3
Loss of a loved one due to Covid-19	No	70.7
	Yes	27.1
	I prefer not to answer	2.2
Frequency of going out in the last month	Never	20.1
	Once a week	50.8
	Several times a week	16.6
	Everyday	11.9
	Many times a day	0.6
Satisfaction about public information	(Five-point Likert scale)	M(*SD*) 2.49 (0.82)
**General health-related information**		**%**
Diagnosed with a chronic disease	No	75.9
	Yes	24.1
Under medical treatment	No	69.6
	Yes	30.4

**TABLE 2:** Descriptive statistics of the scales.

	M (*SD*)	Median	Skewness		Kurtosis	
				
			Statistics	SE	Statistics	SE
GAD-7	6.52 (4.6)	6	1.284	0.137	1.279	0.272
PHQ-9	6.25 (4.3)	5	1.502	0.137	3.263	0.272
PSS-10	18.48 (3.0)	18	-0.614	0.139	3.799	0.277

Correlations between variables are shown in Table 3. We found that younger age was associated with higher anxiety, depression, and perceived stress (*Ps* < 0.001). In addition, the more our participants were worried about the pandemic and concerned about being infected by others, the greater were their symptoms of anxiety (*Ps* < 0.001).

**TABLE 3:** Spearman’s correlations coefficients among continuous variables.

Variables	2	3	4	5	6	7	8	9
GAD-7	0.697***	0.455***	–0.168***	0.239***	0.055	0.208***	0.139	–0.030
2. PHQ-9		0.455***	–0.255***	0.125	0.027	0.121	0.136	–0.036
3. PSS-10			–0.238***	0.067	0.007	0.074	0.059	–0.005
4. Age				0.286***	0.284***	0.105	–0.073	−0.159**
5. Worries about the pandemic					0.348***	0.514***	0.260***	–0.112
6. Risk perception						0.369***	0.303***	–0.116
7. Concern about being infected by others							0.467***	0.042
8. Concern about infecting others								–0.022
9. Satisfaction about public information								

**Ps< 0.01; ***Ps< 0.001.

Mann-Whitney *U*-test and Kruskal-Wallis test analyses revealed several statistically significant differences among participants as regards psychological health. Specifically, compared with men, women reported higher levels of anxiety (*U* = 10.663; *P* < 0.001) and depressive symptoms (*U* = 10.841; *P* < 0.001). Furthermore, group differences related to marital status were detected for all psychological health outcomes, such that unmarried participants reported greater symptoms of anxiety (*H* = 15.358; *P* = 0.002) and depression (*H* = 21.146; *P* < 0.001), and higher perceived stress (*H* = 17.378; *P* = 0.001) than married or cohabitating couples. Participants who reported having children showed lower anxiety (*U* = 9.688; *P* < 0.001) and depression (*U* = 9.283; *P* < 0.001), as well as less perceived stress (*U* = 9.362; *P* = 0.001) than participants without children.

In addition, considering participants’ occupational status, significant group differences emerged on depressive symptoms (*H* = 21.128; *P* = 0.004) and perceived stress (*H* = 25.638; *P* = 0.001). Specifically, retired people reported the lowest levels of depressive symptoms followed by self-employed individuals, whilst the highest levels of depressive symptoms were detected among students with a part-time job. Considering perceived stress, the lowest levels were reported by retired participants and the highest levels by students and students with a part-time job.

Household characteristics (such as having pets, having a balcony or a garden) did not affect psychological outcomes. No significant effects were found when we examined the associations between other Covid-19 related information (such as being diagnosed with Covid-19 and having lost a loved one due to the disease), general health status, and psychological health.

## Discussion and Conclusion

We examined the impact of the Covid-19 outbreak on the psychological health (anxiety and depressive symptoms, and perceived stress) of individuals living in Lombardy, one of the worst hit Italian regions. Specifically, the study was conducted during the final period of the lockdown (i.e., 1 month and a half since the beginning of the pandemic), in which very restrictive measures (including confinement) were enacted by the Government to contain the spread of the virus. Our goal was not only to examine the psychological conditions of the community, but also to identify what factors were related to negative psychological outcomes of such a critical situation, focusing on socio-demographic and housing factors, Covid-19 related aspects, and general health status.

Our findings confirmed that, when the study was conducted, people who lived in Lombardy were worried about Covid-19 (with 92.4% of participants reporting to be from moderate to extremely worried about the pandemic), which confirms results from other Italian studies suggesting that the concerns of the community are associated with the geographical proximity to the center of the pandemic (Pagnini et al., 2020). Moreover, in our research, participants’ worries related to Covid-19 (including concerns about being infected by others) were associated with worsened psychological conditions, which corroborates the conclusions of other studies highlighting the negative emotional consequences of the current pandemic (Eisazadeh et al., 2020). Women, unmarried individuals, and students were the most affected groups, with poorer psychological outcomes than the rest of the sample. Overall, the general high levels of concern found in our participants can be partially explained by the fact that most of the respondents (81%) were females. In this regard, a study by Gerhold (2020) showed that women are more likely to be worried about the pandemic than men.

Surprisingly, participants’ younger age was associated with greater worries about the pandemic. This is interesting, especially if one considers that younger people reported worsened psychological conditions in other Covid-19 studies (e.g., Wang H. et al., 2020) and that, based on our findings, students were more distressed than other groups. In this regard, the uncertainty related to the sudden, unexpected transition to distance learning, and concerns about the future (including procedures for assignments and evaluations; see Sahu, 2020) might have played an important role, with negative effects on the psychological health of this younger subgroup of people. It should also be considered that young people may be overrepresented in the other distressed categories identified in our study (unmarried people, people without children, and students). Taken together, our findings suggest that assessing people’s worries and risk perception is important, since these subjective aspects may significantly impact on their psychological conditions and behaviors, also related to the adoption of correct preventive strategies (Khosravi, 2020).

In this study, we also examined the role of housing conditions, with the hypothesis that these factors might have affected the individuals’ psychological health during the lockdown. Surprisingly we did not find any significant effect of housing characteristics, such as house size, having a balcony or a garden. Indeed, there is need for more research to further understand what type of household situations are associated with mental health outcomes when people are confined to their homes during pandemics. This is particularly important considering that other Covid-19 outbreaks are expected in the next future.

Our data suggest that social isolation negatively affects the psychological health of the community, especially among young unmarried individuals without children. As underlined in other studies, people who are more socially connected live longer and healthier than isolated individuals (Umberson and Montez, 2010). In this regard, social support can contribute to increase self-monitor and self-control (Pilcher and Bryant, 2016), which represent important resources while coping with stressful situations. At the same time, conjoint efforts to cope with a stressor as a couple may lead to enhanced couple satisfaction (Molgora et al., 2019). On the other hand, forced cohabitation and greater levels of stress due to the pandemic may increase the risk of domestic violence and abuse (Barbara et al., 2020; Bradbury-Jones and Isham, 2020). This issue is very important and requires further research. Despite the significant number of studies conducted during the Covid-19 outbreak, there is still a gap in the literature regarding how couples and families cope with this stressful situation. Our findings, combined with those from other studies (Saita et al., 2016), suggest that promoting couple adaptive behavioral and emotional coping strategies (i.e., positive dyadic coping) may be particularly useful in situations that entail dealing with a disease (including the threat of a disease, for those who have not been infected by Covid-19).

Besides the interesting findings reported in this article, our study presents several limitations. First, our sample is not fully representative of the general population due to self-selection bias, since most participants were female. Second, the cross-sectional nature of the research design did not allow to investigate adjustment trajectories over time, which is very important, given the rapid changes of the situation.

Despite these limitations, our study contributed to clarify the short-term psychological impact of the disease by identifying individual characteristics associated with more negative psychological outcomes. Therefore, our findings may offer interesting suggestions for future studies and interventions aimed at promoting the psychological health of the community during pandemics.

## Data Availability Statement

The raw data supporting the conclusions of this article are available from the corresponding author on reasonable request.

## Ethics Statement

The studies involving human participants were reviewed and approved by the Ethics Commission of the Psychology Department Catholic University of Sacred Heart. Participants provided electronic informed consent.

## Author Contributions

ES developed the main conceptual idea. SM conducted the statistical analysis. FF completed the literature review and finalized the research questions. ES, FF, and SM took the lead in writing the manuscript. FP provided critical feedback and helped shape the research, analysis and manuscript. All authors conceived and planned the methods and contributed to data collection.

## Conflict of Interest

The authors declare that the research was conducted in the absence of any commercial or financial relationships that could be construed as a potential conflict of interest.

## Footnotes

1 https://www.who.int/news/item/27-04-2020-who-timeline---covid-19
